# Radial extracorporeal shock wave therapy for the management of spasticity in cerebral palsy: a randomized controlled trial with different time intervals between sessions

**DOI:** 10.3389/fneur.2026.1775659

**Published:** 2026-06-05

**Authors:** Míriam Tur Segura, Antoni Morral, Francisca Gimeno Esteve, Tamara Biedermann Villagra, Ana Mangas Hernández, Sonia Ancosta Diez, Jordi Jiménez Redondo, Nicolás García Rodríguez, Raimon Milà Villarroel

**Affiliations:** 1 Department of Physical Medicine and Rehabilitation, Fundació Aspace Catalunya, Barcelona, Spain; 2 School of Health Sciences Blanquerna, Ramon Llull University, Barcelona, Spain; 3 Research Commission, Fundació Aspace Catalunya, Barcelona, Spain

**Keywords:** cerebral palsy, radial extracorporeal shock wave therapy, rESWT, shock wave therapy, spasticity

## Abstract

**Background:**

Spasticity is the most frequent motor disorder in cerebral palsy (CP). Radial extracorporeal shock wave therapy (rESWT) has emerged as an effective, non-invasive, and well-tolerated alternative for the management of clinical manifestations of spasticity in patients with CP. Despite the variability in administration protocols, no study has evaluated whether extending the time interval between sessions allows prolongation of the therapeutic benefit on these clinical manifestations.

**Methods:**

Participants were randomly assigned to three groups. The control group received three rESWT sessions with a one-week interval between sessions, experimental group 1 received three rESWT sessions with a two-week interval between sessions, and experimental group 2 received three rESWT sessions with a four-week interval between sessions. The primary outcome was the between-group difference in changes in stretch angles of the soleus and gastrocnemius muscles, assessed using the Modified Tardieu Scale. Secondary outcomes were the functional response in overall mobility assessed with the Timed Up and Go Test and improvement in gait assessed with the 10-Meter Walk Test. Outcomes were assessed in all study groups immediately before the first rESWT session and at 3, 5, 9, 12, and 24 weeks from the start of treatment.

**Results:**

Seventy-one patients completed the intervention. In all groups, a significant improvement in passive range of motion and in the catch angle of the triceps surae muscle was observed between baseline and the end of follow-up (*p* < 0.001), with no significant differences between groups and no significant group–time interaction. Age was significantly associated with treatment response, with greater effectiveness observed in younger patients (*p* < 0.001). Regarding secondary outcomes, all groups showed statistically significant improvements in functional tests; however, no differences were found between groups and no significant group–time interaction was observed.

**Discussion:**

Although no significant differences were observed between treatment groups and increasing the time interval between sessions was not associated with a prolongation of the therapeutic effect, all groups showed a statistically significant improvement compared with baseline.

**Conclusion:**

These results suggest that, from a clinical perspective, rESWT application may allow a degree of flexibility, enabling adjustment of the intersession interval according to clinical needs and patient availability without compromising the observed therapeutic effects.

**Clinical trial registration:**

ClinicalTrials.gov, Identifier: NCT05702606.

## 1 Introduction

Cerebral palsy (CP) is a neurodevelopmental condition defined as a permanent disorder of movement and posture that causes activity limitations as a consequence of a non-progressive brain injury occurring in early stages of development (1). CP remains the most common cause of permanent physical disability with onset in childhood and has an estimated prevalence of 17 million people worldwide (2), 1.6 per 1,000 live births in high-income countries, and up to 3.4 per 1,000 live births in low- and middle-income countries (3). The clinical presentation of CP is heterogeneous, reflecting variability in the timing, location, and extent of brain injury, as well as in associated musculoskeletal and neurological impairments (1). Among the motor disorders associated with CP, spasticity is the most prevalent (4).

From a contemporary perspective, spasticity is better conceptualized as a disorder of sensorimotor control following an upper motor neuron lesion, expressed as intermittent or sustained involuntary muscle activation rather than solely a velocity-dependent stretch reflex phenomenon (5). Clinically, the increased resistance perceived during passive muscle stretch (“hyper-resistance”) reflects an interaction between neural contributors (velocity-dependent stretch hyperreflexia and involuntary background activation) and non-neural muscle tissue properties (e.g., elasticity/viscosity and muscle shortening), which is essential when interpreting the treatment effects (6). In individuals with CP, spasticity represents a key component of the multifaceted motor disability and, although it does not appear to be the primary factor interfering with participation, activity, and quality of life (7), it may play an important role by limiting mobility, impairing limb function, causing pain secondary to muscle spasms that may disrupt sleep, and, in the long term, leading to fixed contractures and musculoskeletal deformities (8–10).

The management of spasticity is complex and requires a multidisciplinary assessment, with interventions tailored to individual patient characteristics (2, 4). A wide range of treatment options is currently available, including physiotherapy programs, pharmacological interventions, orthopedic interventions, and surgical options (11), all of which aim to support motor development, improve mobility and dexterity, reduce pain, minimize contracture and deformity, and facilitate the use of orthotic devices, with the ultimate goal of maximizing patient potential and promoting independence and quality of life (2, 10, 11). Despite the diversity of available treatment options, research continues to explore interventions that may contribute to its management. Among these, extracorporeal shock wave therapy (ESWT) has become established in recent years as an effective, non-invasive, non-pharmacological, and low-risk intervention for the management of spasticity-related muscle hyper-resistance. Several studies have demonstrated its favorable effects in the management of clinical manifestations of spasticity in patients with CP, stroke, multiple sclerosis, traumatic brain injury, and spinal cord injury (12–17).

Although the mechanisms of action of ESWT and its effects on muscle hyper-resistance remain incompletely understood, available evidence suggests that ESWT induces multiple biological responses at the peripheral tissue level. These include the induction of nitric oxide production, which may modulate local neuromuscular signaling; activation of biological cascades involving the expression of growth factors related to angiogenesis, resulting in antifibrotic effects through increased blood flow and improved tissue regeneration (18); as well as transient alterations at the neuromuscular junction, such as changes in acetylcholine receptor integrity and motor end plate function, which may temporarily influence muscle activation (19).

There are two types of extracorporeal shock wave therapy: focused (fESWT) and radial (rESWT). These two ESWT types differ in their physical properties, modes of generation, magnitudes of the applied parameters, and depths of penetration. Both are single acoustic impulses with an initial positive peak pressure of approximately 11 megapascals (MPa) in rESWT and more than 100 MPa in fESWT, reached in <1 μs. The pressure produced by fESWT increases rapidly and energy is absorbed to a depth of up to 12 cm, whereas rESWT reaches depths of 3–4 cm (20).

Current literature suggests that treatment with rESWT is effective in reducing clinical manifestations commonly attributed to spasticity in individuals with CP. Most studies have focused on the triceps surae or plantar flexor muscles, with reported effects persisting for up to 12 weeks after treatment. Improvements have been observed in clinical measures of stretch hyper-resistance, including the Modified Ashworth Scale and the Modified Tardieu Scale, together with gains in passive range of motion (pROM), Gross Motor Function Measure (GMFM), and gait and balance parameters, among other functional outcomes. Given that rESWT is a relatively recent approach in the management of spasticity-related manifestations, there is significant variability in administration protocols, particularly regarding the number of sessions per treatment, ranging from a single session to 12 sessions, and the time interval between sessions (21–32). One of the most extensively studied protocols consists of three rESWT sessions with a one-week interval between each session (21, 26, 30, 32). In addition to this variability, few studies have conducted follow-up beyond 12 weeks, and the effect of rESWT using intersession intervals longer than 1 week has not been investigated to determine whether its therapeutic effects can be prolonged over time. In this context, the aim of this study was to evaluate whether increasing the time interval between rESWT sessions could prolong the therapeutic benefits on spasticity-related clinical manifestations in patients with CP.

## 2 Materials and methods

### 2.1 Study design

This study was designed as a randomized, controlled, single-center clinical trial with three parallel groups. The study followed the CONSORT guidelines for non-pharmacological clinical trials, was approved by the FIDMAG Clinical Research Ethics Committee (Barcelona) under reference number PR-2021-16, was conducted in accordance with the World Medical Association Declaration of Helsinki, and was registered at ClinicalTrials.gov (NCT05702606).

The aim of this study was to evaluate whether increasing the time interval between rESWT sessions could extend the therapeutic benefits on the clinical manifestations of spasticity in patients with CP. The specific objectives were: (a) to evaluate whether the effects of the time factor result in an improvement in the muscular clinical manifestations associated with spasticity assessed using the Modified Tardieu Scale; (b) to evaluate whether the effects of the time factor result in an improvement in functional gait response and overall patient mobility; (c) to analyze whether patient age influences treatment outcomes; (d) to assess patient satisfaction with the different shock wave interventions; (e) to assess patients’ perception of pain during application across the different interventions; and (f) to record potential adverse effects associated with the interventions.

#### 2.1.1 Patient and public involvement

Patients and the public were not involved in the design, conduct, reporting, or dissemination of this research.

### 2.2 Participants

Seventy-two patients were recruited from Fundació Aspace Catalunya, a non-profit organization recognized in Spain as a leader in the treatment of neurodevelopmental disorders and CP through person-centered and technologically innovative care. Rehabilitation physicians at the organization selected patients eligible for the study according to the following inclusion criteria: (a) diagnosis of spastic CP; (b) both sexes; (c) age between 4 and 45 years; (d) spasticity in the triceps surae muscle; (e) Gross Motor Function Classification System level I or II; and (f) provision of written informed consent by the participant or their legal guardian. Exclusion criteria were: (a) receipt of shock wave therapy and/or botulinum toxin treatment, and/or focal intramuscular treatment with phenol or alcohol in the triceps surae or any other lower limb muscle within the 6 months preceding the study; (b) any contraindication to the specific treatment (e.g., coagulopathies, malignant tumors in the treated area); (c) history of surgical intervention for orthopedic foot deformities within the previous year; (d) fixed deformities of the ankle joint; and (e) clinical signs of myopathy or neuropathy.

A detailed description of the participant recruitment process is provided in the published study protocol (33).

#### 2.2.1 Randomization

After participants and/or their legal guardians provided written informed consent, they were randomly assigned using an equal-probability algorithm implemented in the R statistical software to one of three intervention groups: control group (CG), *N =* 24; experimental group 1 (EG1), *N =* 24; and experimental group 2 (EG2), *N =* 24. The wide age range for participant inclusion was addressed by stratifying the sample into three age groups: 4–15 years, 16–30 years, and >30 years. The sample was also stratified according to Gross Motor Function Classification System (GMFCS) level.

### 2.3 Intervention

**Figure 1:**
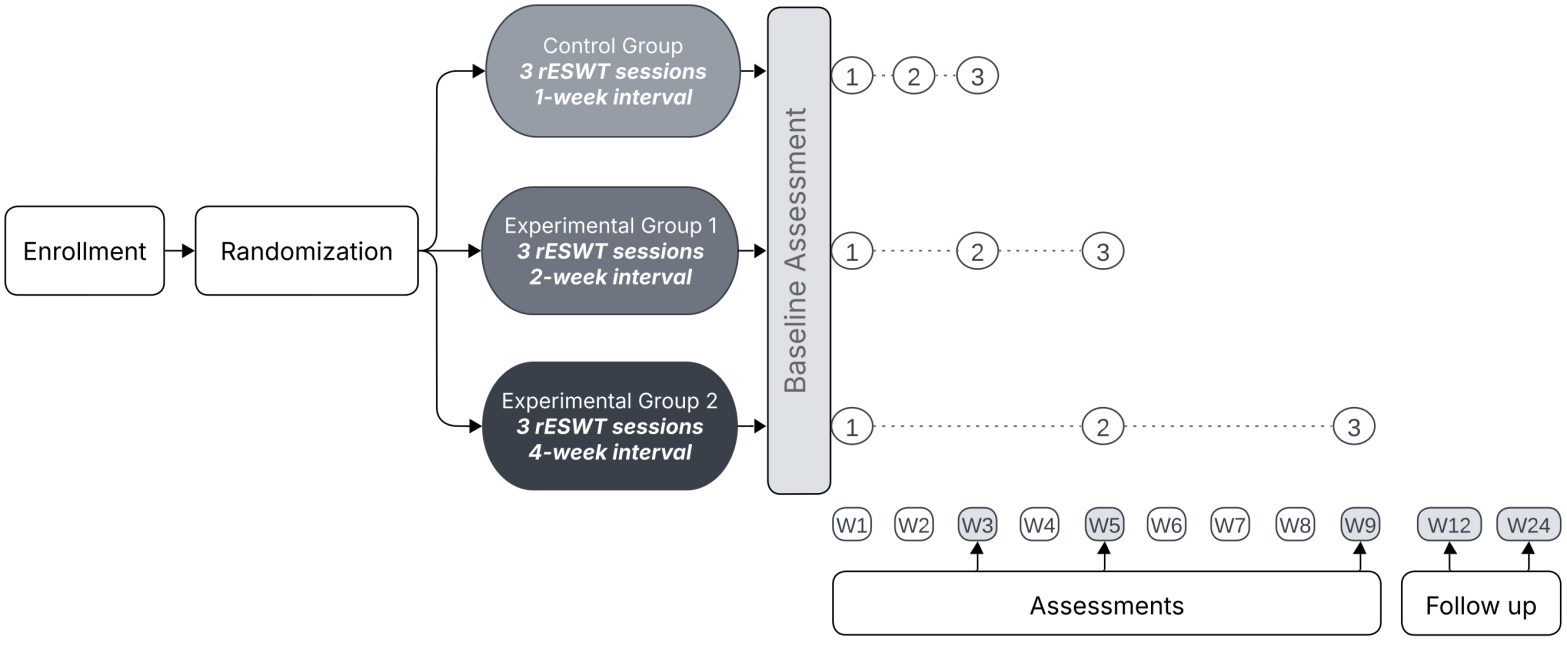
Overview of the study design.

All study participants received three sessions of rESWT. The CG received three sessions with a 1-week interval between sessions, EG1 received three sessions with a 2-week interval between sessions, and EG2 received three sessions with a 4-week interval between sessions (Figure 1).

In each session and for all groups, 2,000 pulses were applied to the treated triceps surae muscle (distributed between the soleus and gastrocnemius muscles) at a pressure of 2.2 bar and a frequency of 8 Hz. In patients with bilateral involvement, treatment was administered to both limbs. Application duration ranged between 4 and 5 min per treated limb. During treatment, patients were positioned prone on the treatment table. Conductive gel was applied to the treatment area, and rESWT was delivered to the muscle belly, with pulses evenly distributed throughout the entire triceps surae region. rESWT application did not require analgesia, sedation, or anesthesia.

The same device was used for all treatment groups: the Swiss DolorClast Smart (EMS Electro Medical Systems) equipped with an EVO Blue handpiece and a 15-mm applicator head. This device ensures cavitation at any frequency and complies with all European safety standards required for medical devices (EN 60601–1, Class I, Type BF, IP40; Directive 93/42/EEC). The intervention was delivered by physiotherapists with experience in the management of spasticity and specific training in radial extracorporeal shock wave therapy. All clinicians followed the same standardized treatment protocol and used the same device throughout the study.

During the study and follow-up period, patients did not receive botulinum toxin treatment and/or focal intramuscular treatment with phenol or alcohol in any lower limb muscle. In addition, rESWT was applied exclusively to the triceps surae, with no application to any other lower limb muscle. All participants were recruited from Fundació Aspace Catalunya, where a transversal and individualized rehabilitation approach is routinely applied. Within the framework of this study, physiotherapy programs were neither standardized nor modified, and no specific exercise protocols were established; therefore, each patient continued with their usual rehabilitation program. During the study period, weekly therapy hours and the type of therapeutic exercises performed were recorded. In addition, physical activities performed outside the rehabilitation center (e.g., gym-based exercise, sports participation, or recreational physical activity) were recorded, including activity type and weekly duration. At each assessment visit, any changes in the intensity, frequency, or type of therapeutic exercise compared with baseline were recorded.

### 2.4 Clinical outcome measures

Outcome measures were collected in all participants at six time points during the study: at baseline, immediately before the first rESWT session (BA); at 3 weeks (3 W); at 5 weeks (5 W); at 9 weeks (9 W); at 12 weeks (12 W); and at 24 weeks from the start of treatment (24 W).

#### 2.4.1 Primary outcome measure

The primary outcome of this study was the between-group difference in changes in joint angles of the soleus and gastrocnemius muscles, assessed using the Modified Tardieu Scale (MTS). This scale evaluates muscle response to passive stretch applied at different velocities. Slow-velocity stretch (V1) allows identification of the angle of maximal muscle length without reflex activation, termed R2, which represents passive range of motion (pROM). Fast-velocity stretch (V3) elicits activation of the stretch reflex and allows identification of the R1 angle, corresponding to the point of sudden resistance or “catch.”

The MTS may be more sensitive than other clinical measures for detecting velocity-dependent stretch responses, and its inter- and intra-observer reproducibility is acceptable (34). All measurements were performed using the same inclinometer to determine the angular values of the stretches at both velocities. For assessment, the patient was positioned supine on the examination table, and separate stretches were applied for the gastrocnemius muscle (measured with the knee extended) and the soleus muscle (measured with the hip and knee flexed at 90°). The inclinometer was placed on the surface of the fifth metatarsal. A value of 0° was defined as 90° of ankle flexion, corresponding to the anatomical position of the foot in standing; degrees exceeding the 0° position were recorded as positive values, and those not reaching this position were recorded as negative values.

#### 2.4.2 Secondary outcome measures

Functional response in the improvement of overall patient mobility was assessed using the Timed Up and Go Test (TUG), in which the time required for the participant to stand up from a chair, walk 3 meters, turn, return, and sit down again with the back supported against the chair was measured. Time was recorded in seconds using a stopwatch. All patients performed the test twice, with a one-minute rest between attempts, and were instructed to “perform the test as fast as possible without running.” The best recorded time was documented for analysis. All participants wore their usual footwear during this test (35).

Functional response in gait improvement was assessed using the 10-Meter Walk Test (10MWT), in which the time required for the participant to walk 10 meters in a straight line was recorded. Time was measured in seconds using a stopwatch. All participants performed the test twice, with a one-minute rest between attempts, and were instructed to “walk as fast as possible without running.” The best recorded time was documented for analysis. All participants wore their usual footwear during this test (36).

#### 2.4.3 Harms

Adverse events were monitored throughout the study period using non-systematic (passive) surveillance. Participants and/or their legal representatives were asked to report any adverse effects during and after each intervention session, and treating clinicians documented any adverse events observed during treatment sessions or follow-up visits. No adverse effects related to the intervention were documented, apart from transient skin erythema that resolved spontaneously within a few minutes.

### 2.5 Sample size

Sample size was calculated using the GRANMO software, accepting an alpha risk of 0.05 and a beta risk of 0.2. A total of 24 participants per group, resulting in 72 patients overall, was required to detect a difference of 5 degrees or greater in R1 and R2 of the MTS. A common standard deviation of 16.44 was assumed, with a correlation coefficient of 0.91 between baseline and final measurements. These parameters were derived from a study on the treatment of spasticity using shock wave therapy in patients with CP (26). A loss to follow-up rate of 10% was estimated.

### 2.6 Assessor blinding

In this study, the same assessing physiotherapist and assistant, both with extensive experience in the field, conducted all outcome assessments and remained blinded to group allocation throughout the intervention period. Interventions and assessments were carried out in different locations within the center to maintain assessor blinding, and the data analyst was also blinded to treatment allocation. Allocation concealment was centralized and managed by one of the investigators, who informed participants and/or their families of the assigned intervention by telephone. Allocation information was recorded in the corresponding section of the case report form and was not accessible to the assessing physiotherapist or their assistant at any time during the study. Given the nature of the intervention, blinding of participants and treating physiotherapists was not feasible.

### 2.7 Statistical analysis

A descriptive statistical analysis was performed to characterize and compare variables across the study groups. Prior to descriptive analyses, data normality was assessed using the Shapiro–Wilk and Kolmogorov–Smirnov tests, as well as normality plots (Q–Q plots). Based on the results of these assessments, parametric tests were selected for subsequent analyses. Descriptive statistics included measures of central tendency and dispersion, such as mean and standard deviation for quantitative variables, and absolute frequencies and percentages for qualitative variables.

For longitudinal outcomes (MTS, TUG, and 10MWT), linear mixed-effects models were applied to appropriately account for the repeated-measures structure of the data and within-subject correlation across follow-up sessions. Group (three levels), time (six follow-up sessions), and the group-by-time interaction were specified as fixed effects. A random intercept for participants was included to model individual variability. Age was incorporated as a prespecified covariate to adjust for its potential confounding effect. Model-based inference was derived from the global fixed effects, with primary emphasis on the group effect and the group-by-time interaction. In the absence of statistically significant interaction terms, no *post-hoc* pairwise comparisons between time points were performed. Model performance was evaluated by assessing the significance of fixed effects using Wald tests, and assumptions of homoscedasticity and normality of residuals were verified using residual plots and appropriate tests. For pain perception during treatment and therapy satisfaction only two assessments (baseline and post-intervention) were available. These variables were analyzed using one-way analysis of variance (ANOVA) to compare between-group differences in change scores. When the overall F-test was statistically significant, pairwise *post-hoc* comparisons were conducted using Bonferroni adjustment to control for family-wise error rate. Assumptions of normality and homogeneity of variance were assessed prior to analysis. Statistical significance was set at *p* < 0.05. Data analyses were performed using SPSS software and the lme4 package in R software version 4.5.1 for model fitting.

## 3 Results

**Figure 2:**
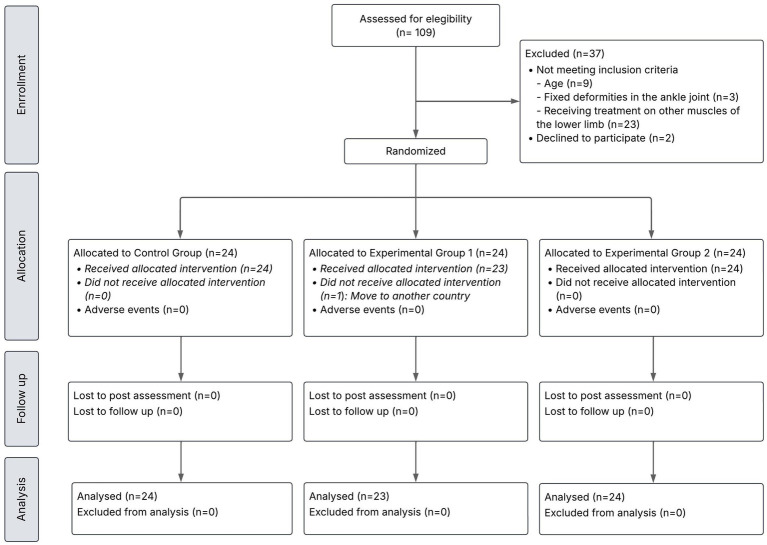
Participant flow diagram.

Of a total of 109 potential participants, 37 were excluded: 9 were outside the eligible age range, 3 presented fixed deformities of the ankle joint, 23 were receiving treatment with botulinum toxin A (BoNT/A) and/or rESWT in other lower limb muscles, and 2 declined to participate in the study. A total of 72 participants provided written informed consent and were included in the study. One participant withdrew after randomization and before initiation of the intervention due to relocation to another country. A total of 71 participants completed the study and follow-up and were subsequently analyzed. A participant flow diagram is presented in Figure 2. Patient recruitment began on October 2021, and follow-up of the last patient was completed on March 2023. No participants were lost between the start of the intervention until the culmination of the follow-up.

Baseline characteristics of the study participants are shown in Table 1. No significant differences were observed among the three groups at baseline with respect to participant characteristics, including age, sex, GMFCS level, and weekly therapeutic exercise load.

**Table 1:** Baseline participant characteristics.

Characteristic	CG (*N =* 24)	EG1 (*N =* 23)	EG2 (*N =* 24)
Sex, *n* (%)			
Male	13 (54)	15 (65)	13 (54)
Female	11 (46)	8 (35)	11 (46)
Age			
Mean (SD)	15.04 (9.41)	15.78 (11.15)	17.38 (11.64)
GMFCS, *n* (%)			
I	18 (75)	18 (78)	15 (63)
II	6 (25)	5 (22)	9 (38)
Therapeutic exercise, hours per week			
Mean (SD)	1.93 (1.28)	1.86 (1.58)	2.05 (1.21)

CG, control group; EG1, experimental group 1; EG2, experimental group 2; GMFCS, Gross Motor Function Classification System; SD, Standard Deviation.

### 3.1 Primary outcome

**Figure 3:**
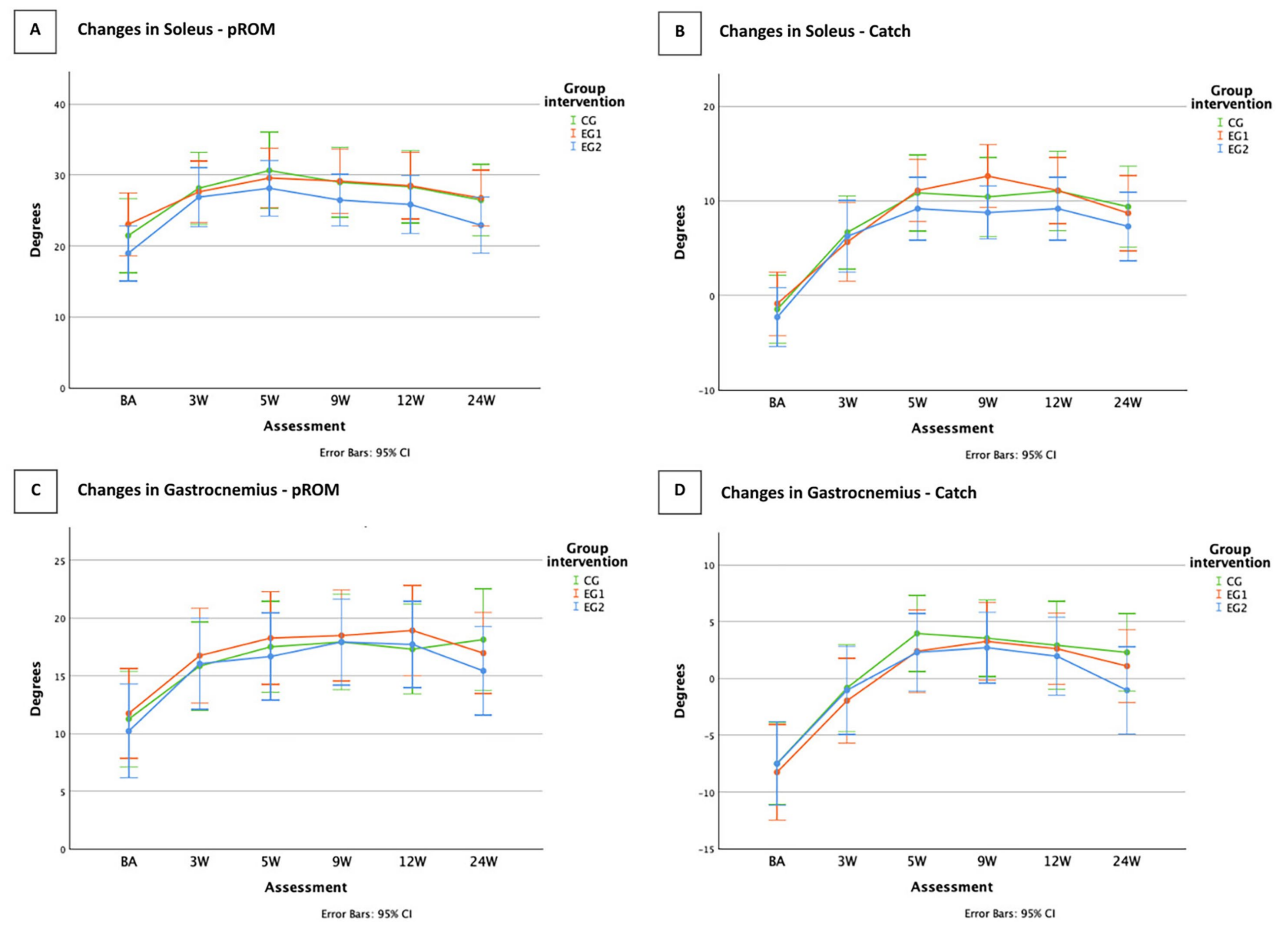
Primary outcome results: changes in modified tardieu scale.

In the assessment of passive range of motion (pROM) of the soleus muscle, no significant differences were observed between groups (*p* = 0.669), nor for the interaction between time and group (*p* = 0.417). A significant main effect of time was detected (*p* < 0.001), indicating an overall improvement across the follow-up period. As the group-by-time interaction was not significant, the pattern of change over time was comparable between groups. Therefore, results are presented as overall longitudinal changes rather than pairwise comparisons between specific time points. All groups showed improved outcomes at 24 weeks compared with baseline: CG (21.46° vs. 26.46°), EG1 (22.83° vs. 26.09°), and EG2 (18.96° vs. 22.92°) (Table 2 and Figure 3A).

**Table 2:** Primary outcome: modified Tardieu scale.

Modified Tardieu scale	Assessment	CG	EG1	EG2	*p*-value	*p*-value group	*p*-value time × group
		Mean (SD)	Mean (SD)	Mean (SD)			
Soleus pROM degrees (R2)	BA	21.46 (12.38)	22.83 (10.43)	18.96 (9.21)	< 0.001	0.669	0.417
	3 W	28.13 (12.05)	27.17 (10.53)	26.14 (9.87)			
	5 W	30.63 (12.80)	28.91 (10.44)	28.12 (9.19)			
	9 W	28.57 (11.63)	28.48 (11.22)	26.46 (8.66)			
	12 W	28.33 (12.13)	28.25 (11.50)	25.87 (9.85)			
	24 W	26.46 (11.93)	26.09 (9.41)	22.92 (9.43)			
Soleus “Catch” degrees (R1)	BA	−1.46 (8.53)	−0.87 (7.78)	−2.29 (7.37)	< 0.001	0.705	0.733
	3 W	6.67 (9.17)	5.65 (9.57)	6.36 (9.28)			
	5 W	10.83 (9.52)	11.09 (7.53)	9.17 (7.89)			
	9 W	10.24 (10.30)	12.61 (7.67)	8.75 (6.63)			
	12 W	11.04 (10.00)	11.00 (7.71)	8.91 (7.97)			
	24 W	9.38 (10.14)	8.70 (9.20)	7.08 (8.84)			
Gastrocnemius pROM degrees (R2)	BA	11.25 (9.81)	11.74 (9.00)	10.21 (9.61)	< 0.001	0.901	0.464
	3 W	15.83 (9.05)	16.74 (9.49)	15.91 (9.47)			
	5 W	17.50 (9.33)	18.26 (9.25)	16.67 (8.93)			
	9 W	18.13 (9.42)	19.25 (9.10)	17.92 (8.84)			
	12 W	17.29 (9.21)	18.48 (9.07)	17.61 (9.03)			
	24 W	18.10 (10.41)	16.96 (8.08)	15.42 (9.08)			
Gastrocnemius “Catch” degrees (R1)	BA	−7.50 (8.60)	−8.26 (9.72)	−7.50 (8.72)	< 0.001	0.875	0.791
	3 W	−0.83 (9.05)	−1.96 (8.63)	−0.45 (9.12)			
	5 W	3.96 (7.94)	2.39 (8.38)	2.29 (8.07)			
	9 W	3.57 (7.77)	3.26 (7.92)	2.71 (7.37)			
	12 W	2.92 (9.20)	3.50 (6.51)	1.96 (7.94)			
	24 W	1.88 (8.70)	0.65 (7.73)	−1.04 (9.09)			

CG, control group; EG1, experimental group 1; EG2, experimental group 2; SD, Standard Deviation; pROM/R2, Maximum passive range of motion. R1: sudden resistance during a fast passive stretch (“catch”).

For the assessment of the soleus muscle “catch” angle, no significant differences were observed between groups (*p* = 0.705), nor for the interaction between time and group (*p* = 0.733). However, a significant main effect of time was observed (*p* < 0.001), again indicating a general improvement across follow-up that was consistent across groups. All groups demonstrated better outcomes at 24 weeks compared with baseline: CG (−1.46° vs. 9.38°), EG1 (−0.87° vs. 8.70°), and EG2 (−2.29° vs. 7.08°) (Table 2 and Figure 3B). The greatest improvements were observed at week 5 in all groups.

In the assessment of gastrocnemius pROM, findings were comparable. No significant differences were found between groups (*p* = 0.901) or for the group-by-time interaction (*p* = 0.464). A significant main effect of time (*p* < 0.001) indicated overall improvement throughout follow-up, with similar trajectories across groups. Improvements from baseline to 24 weeks were observed in all groups: CG (11.25° vs. 18.19°), EG1 (11.74° vs. 16.96°), and EG2 (10.21° vs. 15.42°) (Table 2 and Figure 3C).

For the assessment of the gastrocnemius catch, no significant differences were observed between groups (*p* = 0.875) or for the group-by-time interaction (*p* = 0.791). A significant overall effect of time (*p* < 0.001) reflected improvement across follow-up in all groups: CG (−7.50° vs. 1.88°), EG1 (−8.26° vs. 0.65°), and EG2 (−7.50° vs. − 1.04°) (Table 2 and Figure 3D). Peak effectiveness was observed at week 12.

All models were adjusted for age. Age showed a significant independent association with the outcomes (*p* < 0.001), with younger participants demonstrating greater overall improvement. However, no significant interaction between age and group was observed, indicating that the intervention effect did not differ according to age. Therefore, age was retained as an adjustment covariate, and stratified analyses were not considered necessary.

### 3.2 Secondary outcomes

In the functional assessments, linear mixed-effects models showed a significant main effect of time for the TUG (*p* < 0.001), indicating overall improvement from baseline to the end of follow-up. However, no significant between-group differences were observed (*p* = 0.370), and the group-by-time interaction was not statistically significant (*p* = 0.487), suggesting comparable longitudinal trajectories across groups. Therefore, inference was based on the global model estimates rather than on pairwise comparisons between time points. Similarly, for the 10MWT, a significant main effect of time was detected (p < 0.001), reflecting general improvement over the intervention period. No statistically significant group effect (*p* = 0.472) or group-by-time interaction (*p* = 0.845) was observed, indicating that improvements were consistent across groups (Table 3).

**Table 3:** Secondary outcomes: timed up and go test and 10-meter walk test.

Outcomes	Assessment	CG	EG1	EG2	*p*-value	*p*-value group	*p*-value time × group
		Mean (SD)	Mean (SD)	Mean (SD)			
TUG (sec)	BA	6.87 (2.40)	6.25 (1.50)	7.24 (3.59)	<0.001	0.370	0.487
	3 W	6.09 (2.00)	5.76 (1.22)	6.87 (3.66)			
	5 W	5.64 (1.54)	5.44 (1.18)	6.49 (3.30)			
	9 W	6.00 (1.70)	5.45 (1.18)	6.41 (3.13)			
	12 W	5.75 (1.39)	5.33 (0.99)	6.16 (2.78)			
	24 W	5.79 (1.27)	5.44 (1.15)	6.34 (3.27)			
10MWT (sec)	BA	6.44 (1.63)	5.95 (1.31)	6.63 (2.37)	<0.001	0.472	0.845
	3 W	5.87 (1.47)	5.53 (1.21)	6.11 (2.38)			
	5 W	5.56 (1.12)	5.21 (1.10)	5.90 (2.31)			
	9 W	5.80 (1.50)	5.25 (0.96)	5.81 (2.13)			
	12 W	5.51 (1.06)	5.36 (1.28)	5.53 (1.99)			
	24 W	5.71 (1.20)	5.39 (1.16)	6.11 (2.78)			

CG, control group; EG1, experimental group 1; EG2, experimental group 2; SD, Standard deviation; TUG, Timed Up and Go test; 10MWT, 10 Meters Walk Test; sec, seconds.

**Figure 4:**
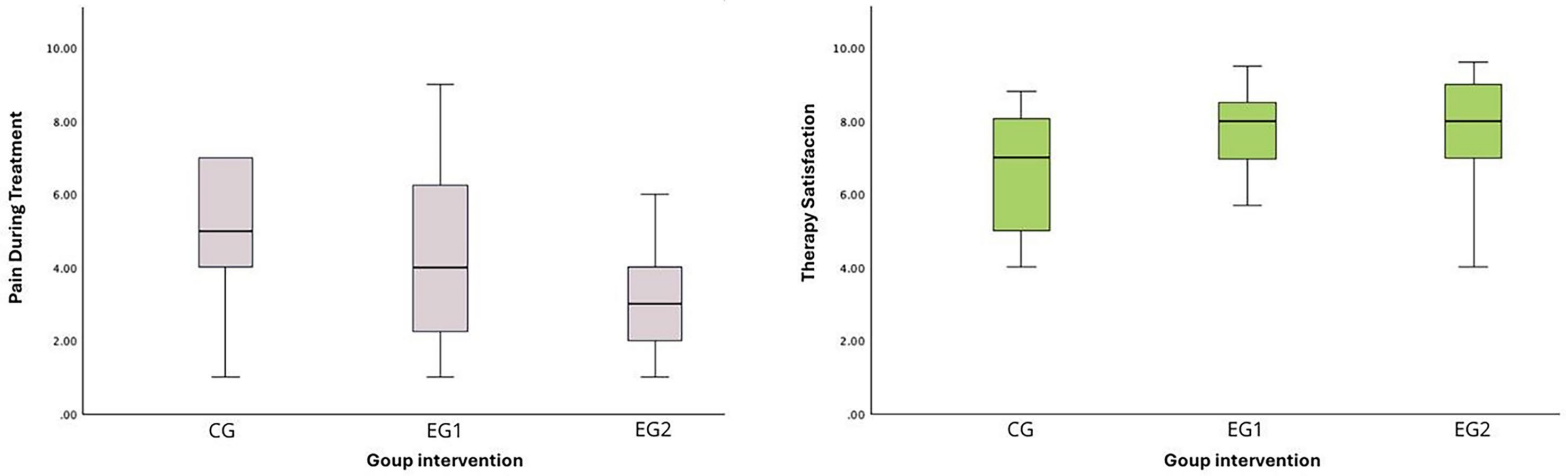
Secondary outcome results: Visual Analogue Scale (VAS) scores for pain during treatment and therapy satisfaction.

Pain during treatment and therapy satisfaction were analyzed as global values aggregated across all sessions. These variables were compared between groups using one-way analysis of variance (ANOVA). Significant group effects were identified for pain perception (*p* = 0.004) and therapy satisfaction (*p* = 0.003). Bonferroni-adjusted *post-hoc* comparisons indicated that EG2 reported significantly lower pain scores compared with the other groups, while both experimental groups reported significantly higher treatment satisfaction compared with the control group (Table 4 and Figure 4).

**Table 4:** Secondary outcomes: VAS scores for pain during treatment and therapy satisfaction.

Outcomes	Group	*N*	Mean	SD	*p*-value
Therapy Satisfaction	CG	24	7.03	1.08	0.003 (CG vs. EG1: 0.002; CG vs. EG2:<0.001)
	EG1	23	8.25	0.77	
	EG2	24	8.33	0.72	
	Total	71	7.86	1.05	
Pain during treatment	CG	24	4.81	1.61	0.004 (CG vs. EG2:0.001; EG1 vs. EG2:0.025)
	EG1	23	4.2	2.26	
	EG2	24	2.83	1.13	
	Total	71	3.94	1.89	

CG, control group; EG1, Experimental Group 1; EG2, Experimental Group 2; SD, Standard deviation.

## 4 Discussion

The data obtained in this study indicate that the time interval between the three rESWT sessions applied to the plantar flexor muscles in patients with CP did not significantly influence the outcomes. All intervention groups showed a very similar evolution of the therapeutic effect across assessment time points, with no statistically significant differences between them. In all three groups, rESWT application produced a progressive improvement in passive joint angle, followed by a stabilization phase and a slight attenuation of the effect between weeks 9 and 24. Results for the fast-stretch “catch” parameter showed a similar pattern, with greater variability between groups but the same temporal evolution: initial improvement, stabilization, and subsequent decline.

For functional gait and global mobility parameters (10MWT and TUG), the trend observed mirrored that of the muscle variables assessed with the MTS. An initial improvement in test execution times was observed, followed by a stabilization phase and a decline at 24 weeks. These findings may suggest that improvements in muscle mechanical properties, in terms of increased extensibility and modulation of velocity-dependent stretch responses, could directly contribute to functional improvements in gait and mobility. This observation had already been documented in a previous quasi-experimental before–after study conducted by our group (30). To date, very few studies have specifically examined the relationship between rESWT-induced changes in muscle function and improvements in task-specific outcomes such as gait or overall mobility relevant to activities of daily living, beyond isolated physiological or parametric changes.

To the best of our knowledge, no study has yet directly compared different intersession intervals of rESWT in patients with CP for the management of spasticity-related clinical manifestations (21–32). Although no significant differences were observed between treatment groups and increasing the intersession interval was not associated with a prolongation of the therapeutic effect, all groups showed a statistically significant improvement compared with baseline throughout follow-up. These results suggest that, from a clinical perspective, rESWT application may allow a degree of flexibility, enabling adjustment of the intersession interval according to clinical needs and patient availability without compromising the observed therapeutic effects.

Although the data from our study indicate an attenuation of the therapeutic effect between weeks 9 and 12, benefits were not completely lost at 24 weeks of follow-up in any of the study groups. This pattern had already been observed in the previous quasi-experimental study (30), which described partial persistence of the clinical effect beyond 12 weeks after intervention. Consistently, Vidal et al. (26), in a crossover study comparing BoNT/A injection with rESWT, reported that at 24 weeks from treatment initiation and after the washout period, improvement in ankle dorsiflexion angles was maintained in the group that had initially received rESWT, but not in the group that had initially received BoNT/A.

A relevant finding of this study is that pediatric patients exhibited a greater therapeutic effect compared with adults, identifying age as a determinant factor in treatment response. To date, published studies on the application of rESWT in CP have not specifically examined treatment effects according to patient age; in studies including wide age ranges, age has generally been used as an inclusion criterion or baseline descriptor, but age-stratified analyses or age–treatment interactions have not been performed. This finding may suggest that structural and functional changes occurring over time in muscles affected by spastic paresis could play a relevant role in treatment response. It is well established that, in CP, muscle anatomy and physiology progressively evolve toward the development of fixed, irreversible deformities and muscle contractures over the years (2). In this regard, a progressive reduction in muscle volume, an increase in non-contractile tissue, and alterations in mechanical properties have been described, with progression related to age and functional severity, together with an unfavorable evolution of the musculotendinous complex associated with insufficient muscle growth and impaired structural adaptability throughout development (37, 38).

From this perspective, during earlier stages of the disorder, muscle tissue may retain greater elasticity and adaptability, facilitating a more evident clinical response to mechanical stimuli such as shock waves. In contrast, in adulthood, myogenic changes and accumulated fibrosis may limit the magnitude of observable change, even in the presence of an effective intervention.

Although the mechanisms of action of rESWT are not yet fully understood, several biological effects at the tissue level have been described, including activation of angiogenesis-related biological cascades, increased microcirculation, and muscle remodeling, which may contribute to an antifibrotic effect (18). In this context, preclinical studies in animal models by Kenmoku et al. (19, 39) have shown that rESWT application can induce transient alterations at the neuromuscular junction, including temporary degeneration and structural modifications of motor end plates, involvement of acetylcholine receptor–related structures, and a transient interruption of neuromuscular transmission, followed by progressive structural and functional recovery over time, without evidence of permanent tissue damage.

Taken together, these mechanisms may contribute to a better understanding of the effects of rESWT on muscle tissue properties in spastic paresis. From a clinical and biological perspective, it is plausible that these effects are more evident when rESWT is applied at stages characterized by greater neuromuscular plasticity and tissue adaptability, before irreversible structural muscle changes become established, as described in CP. This perspective supports the need for future studies to further elucidate the underlying physiological mechanisms and the long-term evolution of rESWT-induced effects.

It is well established that structured rehabilitation programs, including functional training, strengthening, and mobilization, are associated with improved motor and functional outcomes in patients with CP, underscoring the importance of integrated therapeutic approaches (11, 40). In the present study, all participants routinely performed therapeutic physical exercise as part of their daily clinical management. Although physiotherapy programs were not standardized, all patients were treated within the same institution, where a shared transversal and individualized rehabilitation approach is applied. Throughout the study period, weekly therapy hours, the type of therapeutic exercises performed, and physical activities carried out outside the rehabilitation center were systematically recorded, with no relevant between-group differences observed. Within this framework, therapeutic exercise constituted a common component of care and represents the clinical context in which the effects of a physical intervention such as rESWT were evaluated.

Regarding pain perception during rESWT application, although pain perception is a multifactorial phenomenon, significant differences were observed between study groups, with EG2, which received the longest intersession intervals, perceiving the rESWT treatment as less painful compared with CG and EG1. Although direct evidence linking intersession intervals to pain perception in rESWT is currently lacking, the available literature provides a general interpretative framework. In this regard, experimental studies suggest that shock wave application may modulate sensory and nociceptive processing through several processes, including transient alterations in nociceptive transmission, reductions in pain-related neuropeptides, and reversible changes in peripheral sensory nerve fibers (41, 42). These effects are described as dynamic and time-dependent, indicating that sensory responses to shock wave stimulation may vary between applications. From this perspective, longer intervals between sessions may plausibly reduce cumulative sensory stimulation and be associated with better tolerability of subsequent applications. However, this interpretation remains hypothetical and was not specifically evaluated in the present study.

Nevertheless, in all groups, mean pain intensity during application remained below 5 points on the VAS, indicating good overall tolerability. These findings are consistent with those reported by Vidal et al. (26), where rESWT application was associated with low pain levels during the procedure, corresponding to mild discomfort, with values around 4 points on the VAS. Overall, the available literature describes rESWT as a well-tolerated treatment in individuals with CP, consistently reporting the absence of relevant adverse effects and the feasibility of its application without analgesia or sedation, even in young children (22, 23, 25, 26, 28). When patient experience during the session is described, it is usually associated with mild or transient discomfort, without the procedure being perceived as painful or limiting. This good tolerability is further supported by the only qualitative study published to date, conducted by Gimeno et al. (32), which provides direct perspectives from patients and families and describes rESWT as a well-accepted experience associated only with mild discomfort, clearly tolerable, supporting its clinical acceptability as a non-invasive treatment for spasticity management in CP, including pediatric populations.

Regarding treatment satisfaction, both experimental groups reported significantly higher satisfaction levels than the control group. This higher satisfaction may be related to perceived clinical benefit associated with rESWT application, as well as the good tolerability observed across all groups. From a clinical perspective, greater satisfaction with therapy may have relevant implications for treatment adherence and continuity of rehabilitation programs. These results reinforce the acceptance of rESWT as a highly valued option by patients within the multidisciplinary management of spasticity-related impairments.

This study has some limitations that should be considered when interpreting the results. Sample size and the clinical heterogeneity inherent to CP may limit generalizability, although this was addressed through well-defined selection criteria and sample stratification, it remains a factor to consider. Complete blinding of patients and therapists is difficult in physical interventions such as rESWT, which may introduce expectation bias. Variability in rESWT administration protocols limits direct comparison with other studies. Finally, as the intervention was integrated within a rehabilitative approach, it is not possible to fully isolate the specific effect of rESWT, although this reflects routine clinical practice.

Despite the favorable results obtained, substantial work remains to fully understand the biological mechanisms underlying the effects of rESWT on spastic muscle. The use of advanced imaging techniques, biomarkers, and histological analyses may contribute to a better understanding of muscle-level changes induced by this intervention. It is also important for future studies to explore the impact of treatment on participation and daily functioning. Furthermore, standardization of rESWT administration parameters would facilitate comparison across studies and help define more consistent clinical protocols. Finally, multicenter clinical trials with larger samples and longer follow-up periods are needed to strengthen the available evidence and improve the generalizability of results.

## Data Availability

The original contributions presented in the study are included in the article/supplementary material, further inquiries can be directed to the corresponding author.
